# Identification and functional characterization of NAD(P)^+^‐dependent *meso*‐diaminopimelate dehydrogenase from *Numidum massiliense*

**DOI:** 10.1002/mbo3.1059

**Published:** 2020-06-02

**Authors:** Hironaga Akita, Yusuke Nakamichi, Tomotake Morita, Akinori Matsushika

**Affiliations:** ^1^ Research Institute for Sustainable Chemistry National Institute of Advanced Industrial Science and Technology (AIST) Hiroshima Japan; ^2^ Research Institute for Sustainable Chemistry National Institute of Advanced Industrial Science and Technology (AIST) Tsukuba Ibaraki Japan; ^3^ Graduate School of Integrated Sciences for Life Hiroshima University Hiroshima Japan

**Keywords:** d‐Amino acid, meso‐Diaminopimelate dehydrogenase, meso‐Diaminopimelate, NAD(P)^+^‐dependent, Numidum massiliense

## Abstract

*meso*‐Diaminopimelate dehydrogenase (*meso*‐DAPDH) catalyzes the reversible NADP^+^‐dependent oxidative deamination of *meso*‐2,6‐diaminopimelate (*meso*‐DAP) to produce l‐2‐amino‐6‐oxopimelate. Moreover, d‐amino acid dehydrogenase (d‐AADHs) derived from protein‐engineered *meso*‐DAPDH is useful for one‐step synthesis of d‐amino acids with high optical purity. Here, we report the identification and functional characterization of a novel NAD(P)^+^‐dependent *meso*‐DAPDH from *Numidum massiliense* (NmDAPDH). After the gene encoding the putative NmDAPDH was expressed in recombinant *Escherichia coli* cells, the enzyme was purified 4.0‐fold to homogeneity from the crude extract through five purification steps. Although the previously known *meso*‐DAPDHs use only NADP^+^ as a coenzyme, NmDAPDH was able to use both NADP^+^ and NAD^+^ as coenzymes. When NADP^+^ was used as a coenzyme, NmDAPDH exhibited an approximately 2 times higher *k*_cat_/*K*_m_ value toward *meso*‐DAP than that of *meso*‐DAPDH from *Symbiobacterium thermophilum* (StDAPDH). NmDAPDH also catalyzed the reductive amination of corresponding 2‐oxo acids to produce acidic d‐amino acids such as d‐aspartate and d‐glutamate. The optimum pH and temperature for the oxidative deamination of *meso*‐DAP were about 10.5 and 75°C, respectively. Like StDAPDH, NmDAPDH exhibited high stability: it retained more than 75% of its activity after 30 min at 60°C (pH 7.2) or at pHs ranging from 5.5 to 13.0 (50°C). Alignment of the amino acid sequences of NmDAPDH and the known *meso*‐DAPDHs suggested NmDAPDH has a hexameric structure. Given its specificity for both NADP^+^ and NAD^+^, high stability, and a broad range of reductive amination activity toward 2‐oxo acids, NmDAPDH appears to offer advantages for engineering a more effective d‐AADH.

## Graphical abstract

We report the identification and functional characterization of a novel NAD(P)^+^‐dependent *meso*‐DAPDH from *Numidum massiliense* (NmDAPDH). The known *meso*‐DAPDHs use only NADP^+^ as a coenzyme, but NmDAPDH was able to use both NADP^+^ and NAD^+^ as coenzymes.

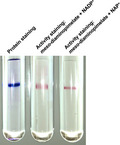

## 1 INTRODUCTION

d‐Amino acids are utilized as important chiral intermediates for agrochemicals, semisynthetic antibiotics, and pharmaceutical drugs. For example, fluvalinate is made from d‐valine, which shows a broad‐spectrum insecticidal activity with a low mammalian toxicity (Chen, Shi, Zhao, Gao, & Zhang, 2016). Daptomycin and Gassericin, which contain a d‐alanine residue in their main body, show an antibacterial activity against several gram‐positive pathogenic bacteria (Cava, Lam, de Pedro, & Waldor, 2011). Also, d‐phenylglycine and d‐*p*‐hydroxyphenylglycine are used as core building blocks of semisynthetic cephalosporins and penicillins (Martínez‐Rodríguez, Martínez‐Gómez, Rodríguez‐Vico, Clemente‐Jiménez, & Las Heras‐Vázquez, 2010). In addition to those applications, d‐glutamic acid is now being used as a cosmetics ingredient (Shiseido 2018). Thus, industrial and pharmaceutical applications are principal contributors to a growing demand for d‐amino acids worldwide (Global Industry Analysts, 2014).

Although d‐amino acids can be produced through both bacterial fermentation and chemical synthesis, the current industrial‐scale method for d‐amino acid production is enzymatic synthesis. In particular, a system utilizing d‐hydantoinase coupled with d‐carbamoylase is considered to be the most proven method at present (Bommarius, Schwarm, & Drauz, 1998; Leuchtenberger et al. 2015). In this system, dl‐hydantoin is initially synthesized from dl‐amino acid, potassium cyanate, and hydrochloric acid through a chemical reaction. Thereafter, d‐hydantoin is selectively hydrolyzed by a stereoselective d‐hydantoinase. Further hydrolysis of the resulting *N*‐carbamoyl‐d‐amino acid to yield the free d‐amino acid is catalyzed by d‐carbamoylase. In addition to this tandem hydrolysis, by coupling hydantoin racemase with the d‐hydantoinase/d‐carbamoylase system, the synthesis is made more efficient. After hydantoin racemase converts l‐hydantoin into d‐hydantoin, the resulting mixture of dl‐hydantoins is used as the precursor for the d‐amino acid. Using this system, several tons of 4‐hydroxy‐d‐phenylglycine have been produced since the 1990s (Schmid, Hollmann, Park, & Bühler, 2002). But while the d‐hydantoinase/d‐carbamoylase/hydantoin racemase system is useful for the synthesis of d‐amino acids and shows high enantioselectivity, it has several drawbacks, including a requirement for multiple chemical and enzymatic reaction steps and the low reaction rate of those enzymes.

To overcome those drawbacks, we previously created a thermostable NADP^+^‐dependent d‐amino acid dehydrogenase (d‐AADH) by introducing Gln154Leu, Asp158Gly, Thr173Ile, Arg199Met, and His249Asn substitutions at the active site of a *meso*‐diaminopimelate dehydrogenase (*meso*‐DAPDH) from *Ureibacillus thermosphaericus* (referred to as UtDAPDH) (Akita, Hayashi, Sakuraba, & Ohshima, 2018). *meso*‐DAPDH is known to catalyze the reversible NADP^+^‐dependent oxidative deamination of *meso*‐2,6‐diaminopimelate (*meso*‐DAP) to produce l‐2‐amino‐6‐oxopimelate (Scapin, Reddy, & Blanchard, 1996). These substitutions in UtDAPDH eliminated its interaction with the l‐amino acid center of *meso*‐DAP, thereby making the enzyme a d‐amino acid dehydrogenase (d‐AADH). The d‐AADH is capable of one‐step synthesis of d‐amino acids and their analogs labeled with stable isotopes through reductive amination of the corresponding 2‐oxo acids (Akita et al., 2018). Moreover, the structural analysis revealed the coenzyme and substrate recognition mechanisms of UtDAPDH, while functional analysis revealed that the specific activity for d‐phenylalanine deamination was enhanced 54‐fold over that of the parent d‐AADH (Akita et al., 2018). Although these findings show that we have overcome the aforementioned limitations of the d‐hydantoinase/d‐carbamoylase/hydantoin racemase system, several drawbacks remain. First, the d‐AADH from *U. thermosphaericus* uses NADP^+^ as the coenzyme, but NAD^+^ is catalytically inert (Akita et al., 2018). When NAD^+^ is used as a coenzyme, the oxidative deamination activity of UtDAPDH and the d‐AADH is not observed by either method of the activity staining or monitoring an increase in absorbance at 340 nm caused by the formation of NADH. These results indicate that UtDAPDH and the d‐AADH cannot use NAD^+^ or NADH as a coenzyme. Meanwhile, utilization of NAD^+^ would be preferable, as it would reduce the cost of d‐amino acid production since NAD^+^ is less expensive. Second, this d‐AADH is unable to catalyze the production of d‐aspartate or d‐glutamate. Thus, the industrial use of this enzyme will require further customization of the coenzyme and substrate specificities.

Here, we report the identification, purification, and functional characterization of an NAD(P)^+^‐dependent *meso*‐DAPDH from *Numidum massiliense* (referred to as NmDAPDH). Also, the overall structure of NmDAPDH and its coenzyme and substrate recognition mechanisms are discussed with a multiple sequence alignment and the homology model.

## 2 MATERIALS AND METHODS

### 2.1 Expression and purification of recombinant protein

The *meso*‐DAPDH gene from *N. massiliense* was designed incorporating *Nde*I and *Eco*RI sites as the 5' and 3' cloning sites and was then constructed by GENEWIZ. The gene was excised from the resulting plasmid using *Nde*I and *Eco*RI and subcloned into pET‐21a (Novagen), yielding pET‐21a/NmDAPDH. *meso*‐DAPDH genes from *Thiobacillus denitrificans* and *Thiobacillus thioparus* were also designed incorporating *Nde*I and *Bam*HI sites as the 5' and 3' cloning sites. After those genes were excised from their plasmids using *Nde*I and *Bam*HI, the resultant genes were subcloned into pET‐16b (Novagen), yielding pET‐16b/TdDAPDH and pET‐16b/TtDAPDH, respectively.

After the three *meso*‐DAPDH derivatives, pET‐21a/NmDAPDH, pET‐16b/TdDAPDH, and pET‐16b/TtDAPDH were separately expressed in *E. coli* BL21 (DE3) cells, the enzymes were purified to homogeneity. The cells were initially grown at 37°C for 3 hr in lysogeny broth medium (500 ml) containing 100 µg/ml ampicillin, after which isopropyl β‐d‐1‐thiogalactopyranoside was added to a final concentration of 0.5 mM, and the culture was incubated for an additional 5 hr. The cells were then harvested, suspended in 50 mM potassium phosphate buffer (pH 7.2), and disrupted by ultrasonication. The resultant lysate was clarified by centrifugation (27,500 g for 15 min at 4°C), after which the supernatant was heated at 55°C for 30 min, and the denatured proteins were removed by centrifugation. The supernatant was applied to a Resource Q column (GE Healthcare) equilibrated with 50 mM potassium phosphate buffer (pH 7.2). The column was then washed with the same buffer, and the enzyme was eluted using an increasing linear NaCl gradient. After the active fractions were pooled, solid (NH_4_)_2_SO_4_ was added to 25% saturation, and the solution was applied to a HiTrap Butyl HP column (GE Healthcare) equilibrated with the same buffer supplemented with 25% (NH_4_)_2_SO_4_. The column was then washed with the same buffer, and the enzyme was eluted using a decreasing linear (NH_4_)_2_SO_4_ gradient. The active fractions were pooled, concentrated using an Amicon Ultra‐15 (Millipore), and loaded onto a Superdex 200 26/60 gel filtration column (GE Healthcare) equilibrated with 20 mM Tris‐HCl buffer (pH 8.0) containing 50 mM NaCl. The active fractions were again pooled and then dialyzed against 10 mM potassium phosphate buffer (pH 7.2). Finally, the dialysate was concentrated using an Amicon Ultra‐15, and the resultant solution was used as the purified enzyme for biochemical experimentation.

### 2.2 PAGE analysis of enzyme

Sodium dodecyl sulfate (SDS)‐PAGE was carried out on a 10% polyacrylamide gel. Precision Plus protein standards (Bio‐Rad Laboratories) were used as the molecular mass standards. The protein sample was boiled for 5 min in 10 mM Tris‐HCl buffer (pH 7.0) containing 1% SDS and 1% 2‐mercaptoethanol. Protein bands were visualized by staining with EzStainAqua.

Native‐PAGE was carried out at 25°C on a 7.5% polyacrylamide gel, after which the protein was stained using EzStainAqua (ATTO). Also, active staining was performed at 50°C using a mixture containing 200 mM potassium phosphate buffer (pH 8.0), 10 mM *meso*‐DAP, 0.1 mM 2‐(4‐iodophenyl)‐3‐(4‐nitrophenyl)‐5‐phenyl‐2*H*‐tetrazolium chloride (Dojindo, Kumamoto, Japan), 0.04 mM 1‐methoxy‐5‐methylphenazinium methyl sulfate (Dojindo), and 1.25 mM NADP^+^ or NAD^+^ until a red band of sufficient intensity was visible.

### 2.3 Enzyme assay

Enzyme activities were measured spectrophotometrically (model UV‐2450; Shimadzu, Kyoto, Japan) by monitoring the increases in the absorbance at 340 nm caused by the oxidative deamination of amino acid, or the decreases caused by reductive amination of 2‐oxo acid. The reaction mixture (1 ml) for the oxidative deamination contained 200 mM glycine‐KOH (pH 10.5), 10 mM *meso*‐DAP, 1.25 mM NADP^+^, and enzyme. The reaction mixture (1 ml) used for the reductive amination contained 200 mM glycine‐KOH (pH 9.0), 200 mM NH_4_Cl (pH 9.0), 5 mM 2‐oxo acid, 0.1 mM NADPH, and enzyme. The extinction coefficient of NAD(P)H was 6.22 mM^−1^ cm^−1^.

Protein concentrations were determined using the method of Bradford (1976). Bovine serum albumin was used as the standard.

### 2.4 Effects of pH and temperature on enzyme activity and stability

The optimal pH values for the oxidative deamination of *meso*‐DAP and the reductive amination of pyruvate were determined at 50°C using 200 mM borate (pH 8.0–9.0), glycine‐KOH (pH 9.0–10.5), and bicarbonate (pH 10.5–11.3) buffers. The optimal temperatures of oxidative deamination of *meso*‐DAP were determined by measuring at temperatures ranging from 50°C to 80°C.

To assess its pH stability, NmDAPDH was incubated for 30 min at 50°C in 100 mM concentrations of the following buffers: succinate (pH 3.5–4.0), acetate (pH 4.0–5.5), citrate (pH 5.5–6.5), phosphate (pH 6.5–8.0), borate (pH 8.0–9.0), glycine‐KOH (pH 9.0–10.5), bicarbonate (pH 10.5–11.3), phosphate (pH 11.3–12.3), and KCl‐KOH (pH 12.3–13.0). The enzyme solutions were then rapidly cooled on ice, and the remaining activities were determined using an oxidative deamination assay. To evaluate its temperature stability, the enzyme was incubated for 30 min at 40–70°C (pH 7.2). The enzyme solutions were then rapidly cooled on ice, and the remaining activities were determined using the oxidative deamination assay.

### 2.5 Determination of kinetic parameters

The initial velocities of the oxidative deamination reactions were analyzed using the standard assay conditions. To determine the kinetic parameters for NADP^+^, NAD^+^, and *meso*‐DAP, several concentrations of NADP^+^ (0.015–1.25 mM), NAD^+^ (0.5–40 mM), or *meso*‐DAP (0.5–15 mM) were used. Using NAD(P)/NAD(P)H assay Kit‐WSTs (Dojindo), it confirmed that NAD(P)H did not include in the NAD(P)^+^ solution. The initial velocities were then plotted against the substrate concentration, and the *K*_m_ and *k*_cat_ values were determined by curve fitting using Igor Pro ver. 3.14 (WaveMetrics).

### 2.6 Homology modeling of NmDAPDH

The homology model structure of NmDAPDH was constructed by Modeller software (Eswar et. al. 2006) using the crystal structure of *meso*‐DAPDH from *Symbiobacterium thermophilum* (referred to as StDAPDH)/NADP^+^/*meso*‐DAP tertiary complex (Liu et al., 2014). Figures depicting molecular structures were generated with an open‐source PyMol molecular graphic system, version 1.8 (Schrödinger).

## 3 RESULTS AND DISCUSSION

### 3.1 Identification and purification of *meso*‐DAPDHs

In our previous study, we showed that interactions between UtDAPDH and NADP^+^ molecules involve fourteen amino acid residues (Akita et al., 2018). In particular, the side chains of Thr35, Arg36, and Arg37, and the backbone nitrogens of Arg36 and Tyr11 form eight hydrogen bonds with the C2‐phosphate group of NADP^+^ molecule. We concluded from those findings that Thr35, Arg36, and Arg37 interact strictly for NADP^+^ recognition. Knowing that, in the present study, we used UtDAPDH as a reference sequence to screen for NAD(P)^+^‐dependent *meso*‐DAPDHs.

**FIGURE 1:**
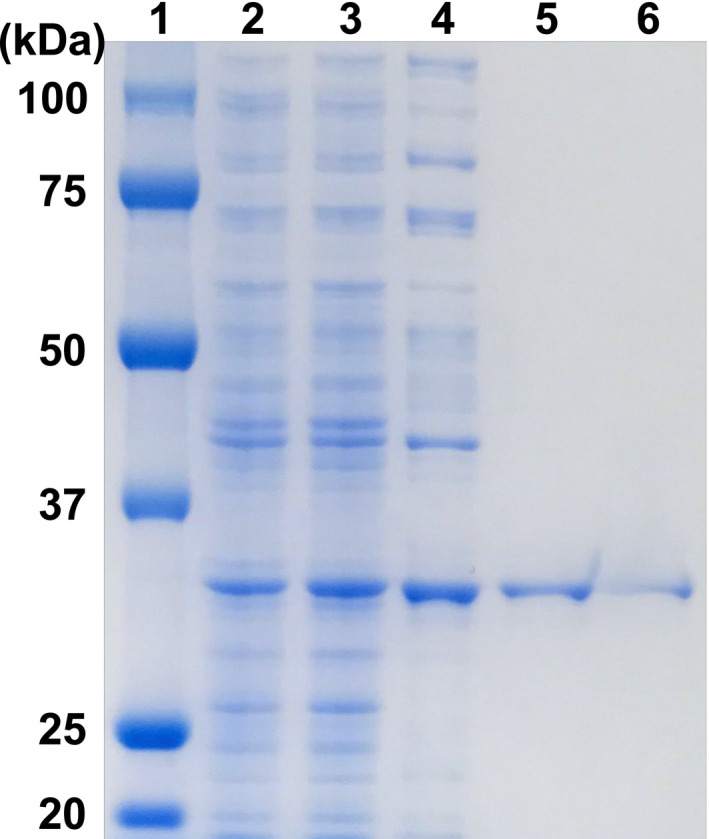
Purification steps followed by SDS‐PAGE. Proteins were separated using SDS‐PAGE and visualized through EzStainAqua staining: lane 1, protein molecular size markers; lane 2, crude extract; lane 3, heat treatment pool; lane 4, Resource Q column chromatography pool; 5, HiTrap Butyl HP column chromatography pool; lane 6, HiLoad 26/60 Superdex 200 pg column chromatography pool

Using the amino acid sequence of UtDAPDH as a reference, a protein BLAST search of the bacterial genome database was carried out to obtain putative *meso*‐DAPDH genes. Among those detected, we focused our attention on three putative *meso*‐DAPDH genes from *N. massiliense*, *T. denitrificans,* and *T. thioparus*. These putative genes did not include the residue corresponding to Thr35 in UtDAPDH, and the three putative proteins shared more than 70% amino acid sequence homology. Anticipating that they likely encode NAD(P)^+^‐dependent *meso*‐DAPDHs, the putative genes were separately overexpressed in *E. coli* BL21 (DE3). However, no soluble proteins were obtained from *E. coli* BL21 (DE3) cells harboring pET‐16b/TdDAPDH or pET‐16b/TtDAPDH. The reason for this remains unclear at present, though preparation of soluble proteins may be improved by using a lower temperature during expression, longer incubation times, a larger amount of culture, and dialysis with a higher concentration of urea (Singh, Upadhyay, Upadhyay, Singh, & Panda, 2015). On the other hand, we obtained a soluble protein from *N. massiliense*, which exhibited oxidative deamination activity toward *meso*‐DAP. After sonication and centrifugation, about 207 mg of soluble protein was obtained from the crude extract. The purified enzyme was isolated using the five‐step procedure described in Materials and methods. The purified NmDAPDH migrated as a single band on SDS‐PAGE (Figure 1), indicating that the enzyme had been purified to homogeneity. At the final step, the enzyme was purified 4.0‐fold with an overall yield of about 1.86%. The specific activity of the purified enzyme toward *meso*‐DAP was 701 ± 0.7 µmol min^−1^ mg^−1^.

### 3.2 Kinetic properties and substrate specificity of NmDAPDH

**FIGURE 2:**
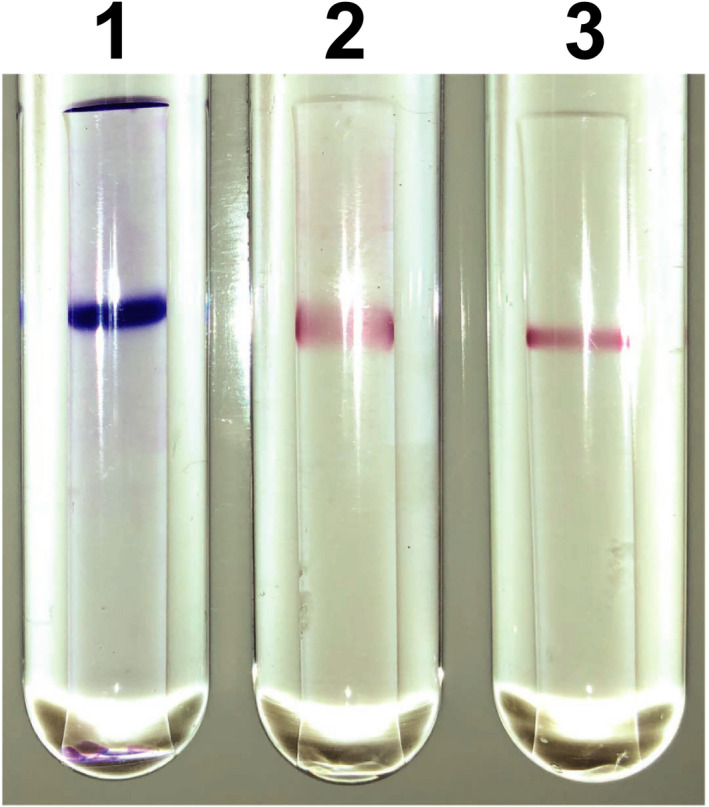
Protein and activity staining of purified NmDAPDH. The purified enzyme was applied to a 7.5% polyacrylamide gel: lane 1, protein staining; lane 2, activity staining with NADP^+^; lane 3, activity staining with NAD^+^

To examine the coenzyme specificity of NmDAPDH, we initially carried out activity staining. After performing native‐PAGE, one clear band was obtained upon protein staining (Figure 2). In addition, the activity staining revealed that purified NmDAPDH was capable of utilizing both NADP^+^ and NAD^+^ as coenzymes. To further evaluate the coenzyme specificity of NmDAPDH, we assessed the kinetic parameters toward NADP^+^ and NAD^+^ as well as *meso*‐DAP (Table 1). The *K*_m_ value toward NADP^+^ indicated its affinity for the enzyme was about 3,000 times higher than that of NAD^+^. Moreover, the *k*_cat_/*K*_m_ value toward NADP^+^ was 260 times higher than that for NAD^+^. These results indicate that NmDAPDH prefers NADP^+^ to NAD^+^. Using NADP^+^ as a coenzyme, the *K*_m_ and *k*_cat_ values toward *meso*‐DAP were calculated to be 3.34 mM and 3.83 × 10^4^ min^−1^, respectively. But given that UtDAPDH uses only NADP^+^ as the coenzyme for the oxidative deamination of *meso*‐DAP, as NAD^+^ is inert (Akita et al., 2018), NmDAPDH appears to have a more relaxed coenzyme specificity than UtDAPDH.

**Table 1:** Kinetic parameters of NmDAPDH and the known *meso*‐DAPDHs with coenzymes and substrate

*meso*‐DAPDH	Coenzyme/Substrate	*k*_cat_ (min^−1^)	*K*_m_ (mM)	*k*_cat_/*K*_m_ (min^−1^·mM^−1^)
*N. massiliense*	NADP^+^	4.75 × 10^4^	0.302	1.57 × 10^5^
	NAD^+^	5.36 × 10^5^	890	6.02 × 10^2^
	*meso*‐Diaminopimelate^c^	3.83 × 10^4^	3.34	1.15 × 10^4^
*S. thermophilum* ^a^	NADP^+^	1.53 × 10^3^	0.35	4.37 × 10^3^
	NAD^+^	N.R.^d^	N.R.	N.R.
	*meso*‐Diaminopimelate^c^	2.43 × 10^3^	0.41	5.93 × 10^3^
Arg71Ala variant of StDAPDH^a^	NADP^+^	3.49 × 10^3^	0.39	8.95 × 10^3^
	NAD^+^	N.R.	N.R.	N.R.
	*meso*‐Diaminopimelate^c^	2.43 × 10^3^	0.42	5.78 × 10^3^
*Bacteroides fragilis* ^b^	*meso*‐Diaminopimelate	4.62 × 10^2^	0.11	4.20 × 10^3^
*Corynebacterium glutamicum* ^b^	*meso*‐Diaminopimelate	6.92 × 10^3^	2.8	2.47 × 10^3^
*Hungateiclostridium thermocellum* ^b^	*meso*‐Diaminopimelate	2.01 × 10^4^	0.21	9.84 × 10^4^
*Lysinibacillus sphaericus* ^b^	*meso*‐Diaminopimelate	9.25 × 10^3^	2.4	3.86 × 10^3^

^a^ Data are from Gao et al. (2017).

^b^ Data are from Xu, Ruan, Liu, Wang, and Zhang (2019).

^c^ Kinetic parameters for *meso*‐diaminopimelate were determined using NADP^+^.

^d^ N.R. means not reported.

We also assessed the reductive amination activity of NmDAPDH. In the presence of NADPH, NmDAPDH showed activity toward several substrates, including 2‐oxooctanoate, oxaloacetate, and pyruvate (Table 2). On the other hand, the specific activity was decreased with NADH (Table 2).

**Table 2:** Substrate specificity of NmDAPDH and StDAPDH

		NmDAPDH		StDAPDH^a^
		NADPH	NADH	NADPH
Substrate	Product	µmol·min^−1^·mg^−1^	µmol·min^−1^·mg^−1^	µmol·min^−1^·mg^−1^
2‐Oxooctanoate	d‐2‐Aminooctanoate	5.8 ± 0.11	N.D.^b^	N.R.^c^
Oxaloacetate	d‐Aspartate	5.2 ± 0.083	0.13 ± 0.0014	N.R.
Pyruvate	d‐Alanine	4.7 ± 0.070	0.13 ± 0.0025	2.9 ± 0.16
4‐Methylthio−2‐oxobutanoate	d‐Methionine	2.0 ± 0.028	N.D.	N.R.
2‐Oxobutanoate	d‐2‐Aminobutanoate	1.4 ± 0.0050	0.011 ± 0.00036	N.R.
4‐Methyl−2‐oxovalerate	d‐Leucine	0.803 ± 0.016	N.D.	0.11 ± 0.03
3‐Methyl−2‐oxobutyrate	d‐Valine	0.26 ± 0.0050	0.0012 ± 0.00047	0.097 ± 0.001
3‐Methyl−2‐oxovalerate	d‐Isoleucine	0.18 ± 0.016	N.D.	N.R.
2‐Oxoglutarate	d‐Glutamate	0.11 ± 0.0042	N.D.	N.R.

^a^ Data are from Gao et al. (2012).

^b^ N.D. means not detected.

^c^ N.R. means not reported.

StDAPDH also catalyzes the oxidative deamination of *meso*‐DAP with NADP^+^ (Gao et al., 2012, 2017). Moreover, StDAPDH shows reductive amination activity toward pyruvate, 4‐methyl‐2‐oxovalerate, and 3‐methyl‐2‐oxobutyrate for the synthesis of d‐amino acids with NADPH (Table 2). When the *k*_cat_/*K*_m_ values toward *meso*‐DAP of StDAPDH (Gao et al., 2017), the Arg71Ala variant of StDAPDH (Gao et al., 2017), and NmDAPDH were compared, the value of NmDAPDH was approximately 2 times higher than that of StDAPDH and the Arg71Ala variant (Table 1). On the other hand, the *k*_cat_/*K*_m_ value toward *meso*‐DAP of NmDAPDH was approximately 9 times lower than that of *meso*‐DAPDH from *Hungateiclostridium thermocellum*, which shows the highest value among the previously known enzymes (Table 1). When NADPH was used as a coenzyme, NmDAPDH showed 1.6‐ to 7.3‐fold higher specific activity toward pyruvate, 4‐methyl‐2‐oxovalerate, and 3‐methyl‐2‐oxobutyrate than those of StDAPDH (Table 2). Also, NmDAPDH showed higher specific activity toward oxaloacetate to produce d‐aspartate than the previously reported values (Zhang et al., 2018). Although StDAPDH has not been tested the NADH‐dependent reductive amination of 2‐oxo acid, NmDAPDH showed the activities toward at least 4 substrates (oxaloacetate, pyruvate, 2‐oxobutanoate, and 3‐methyl‐2‐oxobutyrate) (Table 2). Thus, NmDAPDH has a great potential to create an NAD(P)^+^‐dependent d‐AADH with higher activity than a d‐AADH prepared from StDAPDH.

### 3.3 Effects of pH and temperature on the enzyme activity and stability of NmDAPDH

**FIGURE 3:**
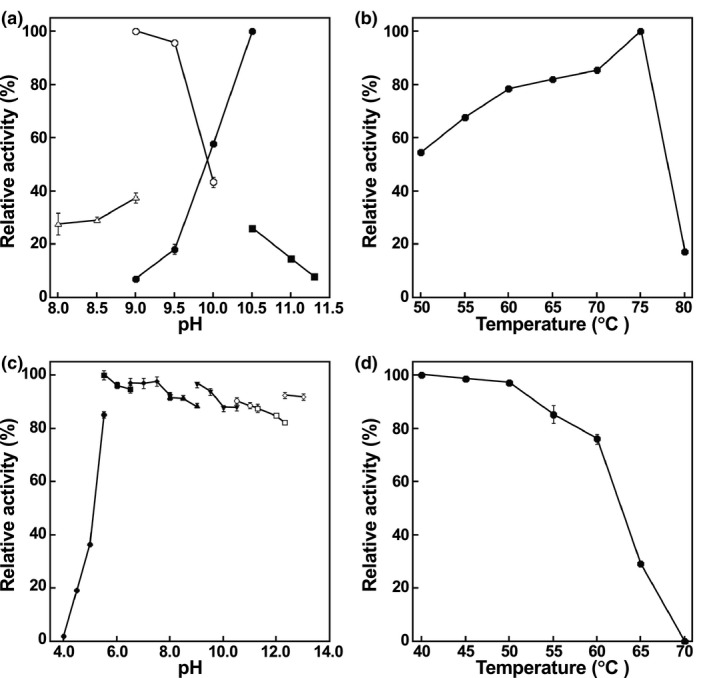
Effects of pH and temperature on NmDAPDH activity and stability. (a) Effect of pH on oxidative deamination of *meso*‐DAP (closed symbols) and reductive amination of pyruvate (open symbols). The buffers used were as follows: triangles, borate; circles, glycineKOH; and squares, bicarbonate. (b) Effect of temperature on oxidative deamination of *meso*‐DAP. (c) Remaining activity toward oxidative deamination of *meso*‐DAP after incubation for 30 min at various pHs (50°C). The buffers used were as follows: closed circles, acetate; closed squares, citrate; closed diamonds, phosphate; closed upward triangles, borate; closed downward triangles, glycine‐KOH; open circles, bicarbonate; open squares, phosphate; and open diamonds, KCl‐KOH. (d) Remaining activity toward oxidative deamination of *meso*‐DAP after incubation for 30 min at various temperatures (pH 7.2). Error bars indicate SE (*n* = 3)

Using NADP^+^ and NADPH as coenzymes, the effect of pH on the oxidative deamination of *meso*‐DAP and the reductive amination of pyruvate catalyzed by NmDAPDH were evaluated. At a temperature of 50°C, the optimum pHs for the oxidative deamination and the reductive amination were 10.5 and 9.0, respectively (Figure 3a). When the temperature dependence at pH 10.5 was examined, maximum activity was observed at 75°C (Figure 3b). We evaluated the effect of pH on the enzyme stability based on the activity remaining after incubation at 50°C for 30 min and found that NmDAPDH retained more than 85% of the activity at pHs between 5.5 and 13.0 (Figure 3c). After incubation for 30 min at various temperatures in 10 mM potassium phosphate buffer (pH 7.2), NmDAPDH retained more than 79% of the activity at 60°C (Figure 3d). On the other hand, no activity was retained at 70°C. These results suggest that the pH stability of NmDAPDH is similar to that of UtDAPDH, which retains more than 80% of the activity at pH 5.0 to 11.0 after incubation for 30 min at 50°C (Akita et al., 2018).

NmDAPDH and the d‐AADH generated through the amino acid substitutions will have several advantages for d‐amino acid production in industrial bioprocesses. Because of their thermostability, these enzymes can be partially purified by heat treatment, which is a simple and effective method to remove a lot of the contaminating proteins derived from *E. coli* cells. Also, because NmDAPDH is stable under the broad range of pHs at 50°C for 30 min, a variety of reaction conditions are available to produce d‐amino acids. Moreover, a higher reaction temperature for d‐amino acid production will prevent contamination of mesophilic bacteria.

### 3.4 Structural elucidation of NmDAPDH

**FIGURE 4:**
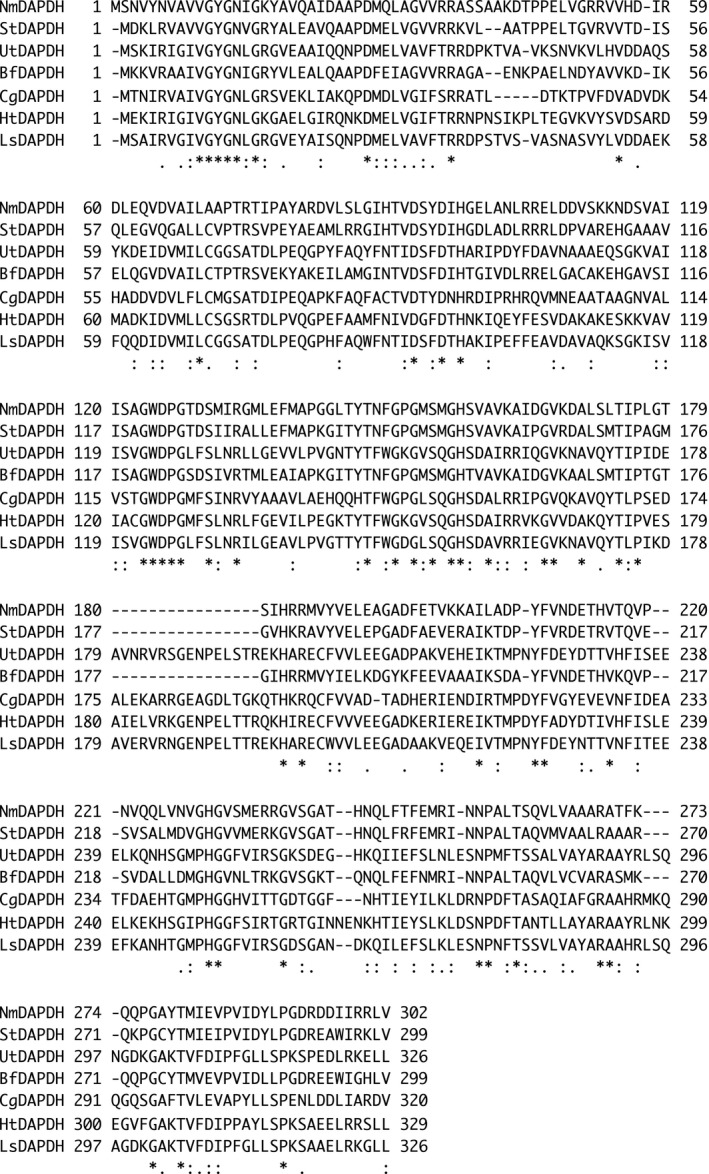
Multiple sequence alignment of *meso*‐DAPDHs from *N. massiliense* (NmDAPDH), *S. thermophilum* (StDAPDH), *U. thermosphaericus* (UtDAPDH), *Bacteroides fragilis* (BfDAPDH), *Corynebacterium glutamicum* (CgDAPDH), *Hungateiclostridium thermocellum* (HtDAPDH), and *Lysinibacillus sphaericus* (LsDAPDH). Fully conserved residues are indicated by asterisks. The residues with strong or weak similarities are indicated by colons and periods, respectively

As mentioned above, the substrate specificity of NmDAPDH is similar to that of StDAPDH. To further elucidate their enzymatic characteristics, an amino acid sequence alignment was prepared with NmDAPDH and the known *meso*‐DAPDHs (Figure 4). The crystal structures of StDAPDH and UtDAPDH have been solved; therefore, their structural features were compared with NmDAPDH. The α9 (residues 177–184) and α10 (residues 193–196) helices in UtDAPDH are included in a dimerization domain and act as key components for formation of a dimeric structure (Akita, Seto, Ohshima, & Sakuraba, 2015). By contrast, α9 and α10 are replaced by a short loop in StDAPDH, which assembles into a hexamer (Liu et al., 2014). Similarly, we found that sequences corresponding to α9 and α10 in UtDAPDH are not conserved in NmDAPDH. We suggest therefore that NmDAPDH likely exhibits a hexameric structure.

**FIGURE 5:**
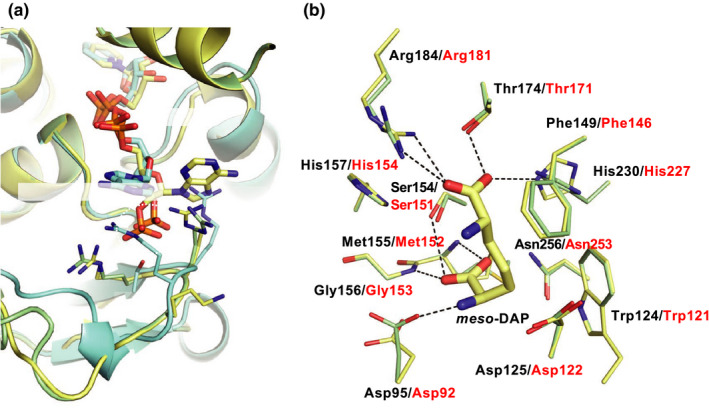
3D structural analysis of NmDAPDH. The NmDAPDH homology model is compared with the crystal structures of StDAPDH/NADP^+^/*meso*‐DAP tertiary complex and UtDAPDH/NADP^+^ binary complex. Atoms are depicted as follows: C in NmDAPDH (green) and C in StDAPDH/NADP^+/^ *meso*‐DAP tertiary complex (yellow), and C in UtDAPDH/NADP^+^ (cyan), O (red), N (blue), and P (orange). Numbers of amino acid residues in NmDAPDH, StDAPDH, and UtDAPDH are shown by black, red, and blue characters, respectively. (a) The superimposed model structures of NmDAPDH, StDAPDH, and UtDAPDH at the binding site of adenine ribose moieties of NADP^+^. (b) The superimposed model structures of NmDAPDH and StDAPDH at the binding site of *meso*‐DAP. The hydrogen bonds in StDAPDH within distances 3.2Å are shown as dashed black lines

When the coenzyme‐binding mechanisms of UtDAPDH and StDAPDH were compared, interactions between the adenine and adenine ribose moieties of NADP^+^ and the enzyme clearly differed (Akita et al., 2015). In the UtDAPDH/NADP^+^ binary complex structure, Thr35, Arg36, and Arg37 interacted strictly with the C2‐phosphate group of the adenine ribose. In StDAPDH, however, those three residues are replaced by Arg35, Arg36, and Lys37, respectively. By these substitutions, the C2‐phosphate group interacts with Arg35 and Arg36, and the adenine ribose of NADP^+^ in the StDAPDH structure is rotated about 80° in a clockwise direction relative to that in the UtDAPDH structure (Akita et al., 2015). Moreover, this rotation in the StDAPDH structure leads to exposing the adenine and adenine ribose moieties of NADP^+^ into the solvent area. In NmDAPDH, Arg35, Arg36, and Lys37 in StDAPDH are replaced with Arg36, Arg37, and Ala38. In other words, amino acid residues (Arg35, Arg36, and Lys37) comprising the C2‐phosphate group‐binding site of StDAPDH are almost conserved in those (Arg36, Arg37, and Ala38) of NmDAPDH. To elucidate the coenzyme recognition mechanism, the crystal structures of StDAPDH/NADP^+^/*meso*‐DAP tertiary complex and UtDAPDH/NADP^+^ binary complex were superimposed on the model structure of NmDAPDH (Figure 5a). The main‐chain conformation of Arg36‐Ala38 in NmDAPDH showed high structural similarity to that of Arg35‐Lys37 in StDAPDH, suggesting that the interactions between the C2‐phosphate group and the enzyme were similar in the NmDAPDH and StDAPDH. By contrast, Ala38 of NmDAPDH and Arg37 of UtDAPDH were arranged at different positions, and UtDAPDH shows that the side chains of Thr35, Arg36, and Arg37 interact with the C2‐phosphate group. Thus, we consider that the molecular mechanism for coenzyme recognition of NmDAPDH is likely similar to that of StDAPDH.

The substrate recognition mechanism was also examined using the amino acid sequence alignment and the homology model. The crystal structure of the StDAPDH/NADP^+^/*meso*‐DAP tertiary complex has been determined (Liu et al., 2014). Within the structure of the complex, *meso*‐DAP interacts with Asp92, Trp121, Phe146, Ser151, Met152, Gly153, Thr171, Arg181, His227, and Asn253. In particular, the hydrophobic interactions between Met152, Asn253, and the substrate play a key role in substrate recognition. The aforementioned residues in StDAPDH are completely conserved in NmDAPDH as Asp95, Trp124, Phe149, Ser154, Met155, Gly156, Thr174, Arg184, His230, and Asn256, respectively (Figure 4). When the tertiary complex of StDAPDH was superimposed on the NmDAPDH model, drastic differences were not observed between both of the enzymes (Figure 5b). This result suggests that the substrate recognition mechanism of NmDAPDH is also likely similar to that of StDAPDH. However, the d‐amino acid recognition mechanism of StDAPDH and NmDAPDH has not been elucidated. To determine the overall structure and elucidate substrate‐binding mechanism of NmDAPDH, we are planning to perform an X‐ray crystallographic analysis of NmDAPDH.

## 4 CONCLUSION

We identified a novel NAD(P)^+^‐dependent DAPDH from *N. massiliense*. NmDAPDH was purified 4.0‐fold to homogeneity from the crude cell extract of *E. coli* expressing the gene encoding the enzyme. The enzyme was capable of utilizing both NADP^+^ and NAD^+^ as coenzymes, though the specificity of NADP^+^ was higher than that of NAD^+^. The *k*_cat_/*K*_m_ value toward *meso*‐DAP of NmDAPDH was approximately 2 times higher than that of StDAPDH. Besides, NmDAPDH was stable at higher temperatures over a wider range of pHs. The thermostability of NmDAPDH under various conditions would be useful for an industrial enzyme. We therefore suggest that with further protein engineering, NmDAPDH has great potential to yield a highly useful d‐AADH for industrial d‐amino acid production.

## CONFLICT OF INTEREST

None declared.

## AUTHOR CONTRIBUTION

Hironaga Akita: Writing‐original draft (lead). Yusuke Nakamichi: Writing‐review & editing (supporting). Tomotake Morita: Writing‐review & editing (supporting). Akinori Matsushika: Writing‐review & editing (supporting).

## ETHICS STATEMENT

None required.

## Data Availability

The *meso*‐DAPDH gene sequences from *N. massiliense*, *T. denitrificans,* and *T. thioparus* are available in the GenBank/EMBL/DDBJ databases under accession numbers MA853437, LDUG01000039, and WP_018507982. The StDAPDH/NADP^+^/*meso*‐DAP tertiary complex and UtDAPDH/NADP^+^ binary complex are available in the Protein Data Bank databases under PDB code 3WBF and 3WYC.
